# Binding of the eukaryotic translation elongation factor 1A with the 5’UTR of HIV-1 genomic RNA is important for reverse transcription

**DOI:** 10.1186/s12985-015-0337-x

**Published:** 2015-08-06

**Authors:** Dongsheng Li, Ting Wei, Hongping Jin, Amanda Rose, Rui Wang, Min-Hsuan Lin, Kirsten Spann, David Harrich

**Affiliations:** Department of Cell and Molecular Biology, QIMR Berghofer Medical Research Institute, Herston, QLD 4029 Australia; School of Biomedical Science, Queensland University of Technology, Brisbane, QLD 4001 Australia; Present address: Centre of Infectious Diseases, Beijing Youan Hospital, Capital Medical University, No. 8 Xitoutiao Youanmenwai, Fengtai District, Beijing, 100069 China

**Keywords:** RNA-protein interaction, eEF1A, HIV-1 genomic RNA, Reverse transcription

## Abstract

**Background:**

The cellular protein eukaryotic translation elongation factor 1A (eEF1A) binds to aminoacylated transfer RNAs and delivers them to the ribosome during translation. eEF1A also binds to RNA secondary structures present in genomes of several RNA viruses and plays important roles in their replication. As a RNA binding protein, whether eEF1A can bind with HIV-1 genomic RNA has not been investigated and was the aim of the study.

**Methods:**

RNA-protein interaction was determined by reversible crosslink co-immunoprecipitation (RC-Co-IP) and biolayer Interferometry assay (BLI). eEF1A binding region within RNA was mapped by deletion and mutation analysis. Virus with genomic RNA mutations were examined for eEF1A-RT interaction by proximity ligation assay, for reverse transcription by qPCR and for replication by CAp24 ELISA in cells.

**Results:**

The interaction of eEF1A with 5’UTR of HIV-1 genomic RNA was detected in cells and *in vitro*. Truncation and substitution mutations in the 5’UTR RNA demonstrated that a stem-loop formed by nucleotides 142 to 170, which encompass a reported tRNA anticodon-like-element, binds to eEF1A. Mutations that altered the stem-loop structure by changing two highly conserved sequence clusters in the stem-loop region result in reduction of the interaction with eEF1A *in vitro*. HIV-1 virus harbouring the same 5’UTR mutations significantly reduced the interaction of eEF1A with HIV-1 reverse transcription complex (RTC), reverse transcription and replication.

**Conclusion:**

eEF1A interacts with 5’UTR of HIV-1 genomic RNA and the interaction is important for late DNA synthesis in reverse transcription.

## Background

The eukaryotic translation elongation factor 1A (eEF1A) is a subunit of the eukaryotic translation elongation 1 complex (eEF1) and its canonical role in translational elongation is to bind and deliver aminoacylated transfer RNAs (aa-tRNAs) to the elongating ribosome. eEF1A is reported to have many moonlighting activities including roles as a protein chaperone, in RNA and actin-binding, protein degradation, nucleocytoplasmic trafficking and multiple aspects of cytoskeletal regulation [1, 2]. There is increasing evidence showing that eEF1A plays a diverse and important roles in the replication of many viruses through the interaction with viral genomic RNA, viral proteins and cellular proteins [1, 3–6].

Previously studies showed that cellular factors are important for efficient HIV-1 reverse transcription [7–11]. Our recent study demonstrated that eEF1A associates with the HIV-1 reverse transcription complex (RTC) and plays an important role in HIV-1 reverse transcription [12]. In newly infected cells where eEF1A is downregulated by transfecting siRNAs, the stability of the viral RTC is greatly decreased, which results in sharply reduced levels of reverse transcription late DNA product [12]. On the other hand, a reported protein: protein interaction between eEF1A and HIV-1 Gag required RNA as a cofactor [13], which was important for virion packaging of eEF1A [13]. However, whether eEF1A can interact with HIV-1 genomic RNA, as it does with other RNA viruses, in order to facilitate association with the HIV-1 RTC has not been investigated.

The HIV-1 genomic RNA 5’UTR has features that could support eEF1A binding as it contains many RNA stem-loop structures and elements including; i) the primer binding site (PBS) which serves as the binding site for cellular tRNA^lys^ [14], and ii) a recently identified tRNA anti-codon-like element (TLE) located proximal to the PBS [15, 16], which can specifically bind to human lysyl-tRNA synthetase [15] and iii) a series of stem-loop structures that regulate polyadenylation, RNA packaging, and RNA dimerization [17]. By using reversible crosslink co-immunoprecipitation (RC-co-IP) and biolayer interferometry (BLI) assay, here we demonstrated that eEF1A binds to the 5’UTR of HIV-1 genomic RNA, and that the binding region within the RNA is located between nucleotides (nt) 106 to 224. Mutations of two highly conserved nucleotide clusters within this RNA sequence, which are predicted to form alternative stem-loop structures, resulted in reduced association of 5’UTR RNA with eEF1A and define nt 142 to 170 as important for interaction with eEF1A. HIV-1 with the same 5’UTR mutations showed a reduced association of eEF1A with reverse transcriptase, inefficient reverse transcription and defective replication in cells. We propose that eEF1A’s role in reverse transcription requires interaction with a conserved stem-loop structure in U5.

## Results

### HIV-1 genomic RNA binds to eEF1A in infected cells

**Fig. 1:**
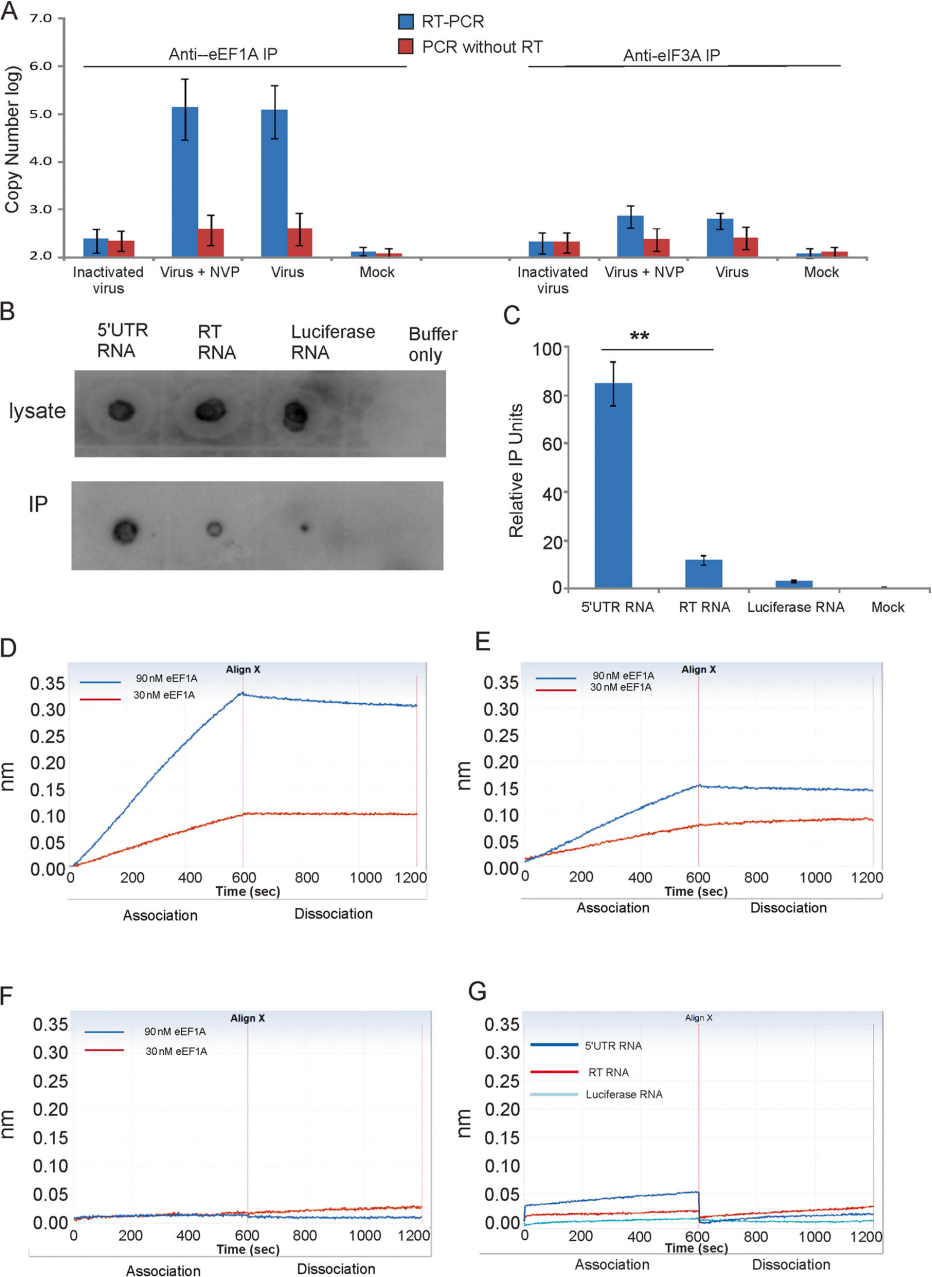
eEF1A binds to HIV-1 genomic RNA. **a** TMZ-bl cells were incubated with HIV-1 for 2 h at 4 °C and 2 h at 37 °C in the presence or absence of nevirapine followed by RC-co-IP using anti-eEF1A or anti-eIF3A antibodies as indicated. The level of RNA extracted from IP product was measured by RT-PCR targeting HIV-1 5’UTR. Data are presented as means ± SD from 3 independent experiments. TMZ-bl cells were also transfected with biotin-labeled RNAs derived from HIV-1 genomic 5’UTR, HIV-1 RT and luciferase sequences followed by RC-co-IP. The RNAs in cell lysates and recovered from IP using anti-biotin antibody (**b**, *upper panel*) or anti-eEF1A antibody (**b**, *lower panel*) were detected by dot blot using streptavidin-peroxidase. The blot signals were quantified using ImageQuant programme and the relative PI unit of IP product to lysate product are presented in **c**. The biotin-labelled **d** 5’UTR, **e** RT or **f** luciferase RNAs were immobilized on the biosensors coupling with streptavidin. The associations of RNAs with eEF1A were measured with OctetRed system using 90nM or 30nM purified eEF1A protein and referenced using 90 nM of BSA in kinetic buffer. The dissociation was measured by moving the biosensor to wells containing kinetic buffer only. **g** The association and dissociation of immobilized 5’UTR, RT and luciferase RNA with 90 nM of eEF1G was also measured as a control. Data are representative of 3 independent experiments. * indicates *p* < 0.05

To examine whether eEF1A interacts with HIV-1 genomic RNA, RC-co-IP experiment was performed in HIV-1 infected TMZ-bl cells using an anti-eEF1A antibody in the presence /absence of an HIV-1 reverse transcriptase inhibitor, nevirapine. Nevirapine is a non-nucleoside RT inhibitor of enzymatic activity. The RNA was extracted from the IP products followed by RT-PCR to examine the levels of HIV-1 RNA. A PCR without reverse transcription reaction was performed as a control to detect DNA contamination. There was a large amount of HIV-1 RNA detected in samples from HIV-1 infected cells compared to infection with heat-inactivated virus (*p* < 0.05), which are defective for viral entry, indicating that HIV-1 RNA co-immunoprecipitated with eEF1A (Fig. 1a). Similar levels of RNA were detected from the infected cells incubated with nevirapine, suggesting that the association of HIV-1 RNA with eEF1A is not dependent on RT activity. Samples analyzed by qPCR, which omit the cDNA synthesis step, showed low levels of viral DNA that were similar to the heat-inactivated samples indicating that the RNA samples were not DNA contaminations (Fig. 1a). Importantly, RC-co-IP using a control antibody anti-eIF3A (Fig. 1a), did not specifically capture HIV-1 RNA suggesting that the interaction between eEF1A and HIV-1 RNA in cells was specific.

### eEF1A directly binds to the 5’UTR of HIV-1 RNA

The association of eEF1A and HIV-1 genomic RNA detected from infected cells could be due to indirect binding through virus proteins. To determine whether eEF1A specifically binds HIV-1 RNA in the absence of the viral proteins, equivalent amounts of biotin-labeled RNAs corresponding to the 5’UTR (the 5’UTR RNA used corresponded to nucleotides 1 to 325 of the HIV RNA genome), a similarly sized RNA from the RT encoding region (RT RNA) or a biotin-labelled luciferase RNA were transfected individually into TZM-bl cells followed by a RC-co-IP assay using an anti-eEF1A antibody. The RNA extracted from the IP products were subjected to a dot blot for biotin labelled RNA detection. As shown in Fig. 1b (upper panel), the RNAs extracted from each lysate contained a similar level of biotin labelled RNA. However a measurably larger amount 5’UTR RNA was recovered from anti-eEF1A co-IP product than RT or luciferase RNAs (Fig. 1b, lower panel and Fig. 1c). The result suggests that 5’UTR RNA interacts with eEF1A in the absence of viral protein.

### 5’UTR RNA binds to eEF1A in biolayer Interferometry (BLI) assay

While co-IP assays can detect associations between molecules, they cannot determine if an association between molecules occurs by direct or indirect binding. To determine if 5’UTR RNA has a direct interaction with eEF1A without any other cellular protein, an *in vitro* BLI assays was employed using purified protein. The BLI method uses the interference pattern of white light reflected from two surfaces on a biosensor probe: a layer of immobilized molecule of interest, such as an RNA or protein, and an internal reference layer. When the biosensor is immersed in a solution containing a molecule of interest, any change in the number of molecules bound to the biosensor tip due to interaction causes a shift in the interference pattern that can be measured. Streptavidin-coated biosensors were saturated with biotin-labelled 5’UTR, RT or luciferase RNAs individually. The association of each RNA with eEF1A was examined using the OctetRed system by incubating each biosensor into kinetic buffer containing 90 nM and 30 nM of eEF1A. The 5’UTR RNA strongly bound with eEF1A at 90 nM (Fig. 1d), while RT RNA showed a weak binding (Fig. 1e), and no binding was detected with luciferase RNA (Fig. 1f). Previously we showed that both eEF1A and eEF1G associated with the HIV-1 RTC [12]. However using BLI assay, no binding was detected between each RNA with 90 nM of eEF1G protein (Fig. 1g), indicating that the association between 5’UTR RNA and eEF1A is specific. The results are consistent with the RC-co-IP results and confirm a direct and specific interaction between 5’UTR of HIV-1 genomic RNA and eEF1A.

### The nt 106 to 224 of 5’UTR RNA is important for interaction with eEF1A

**Fig. 2:**
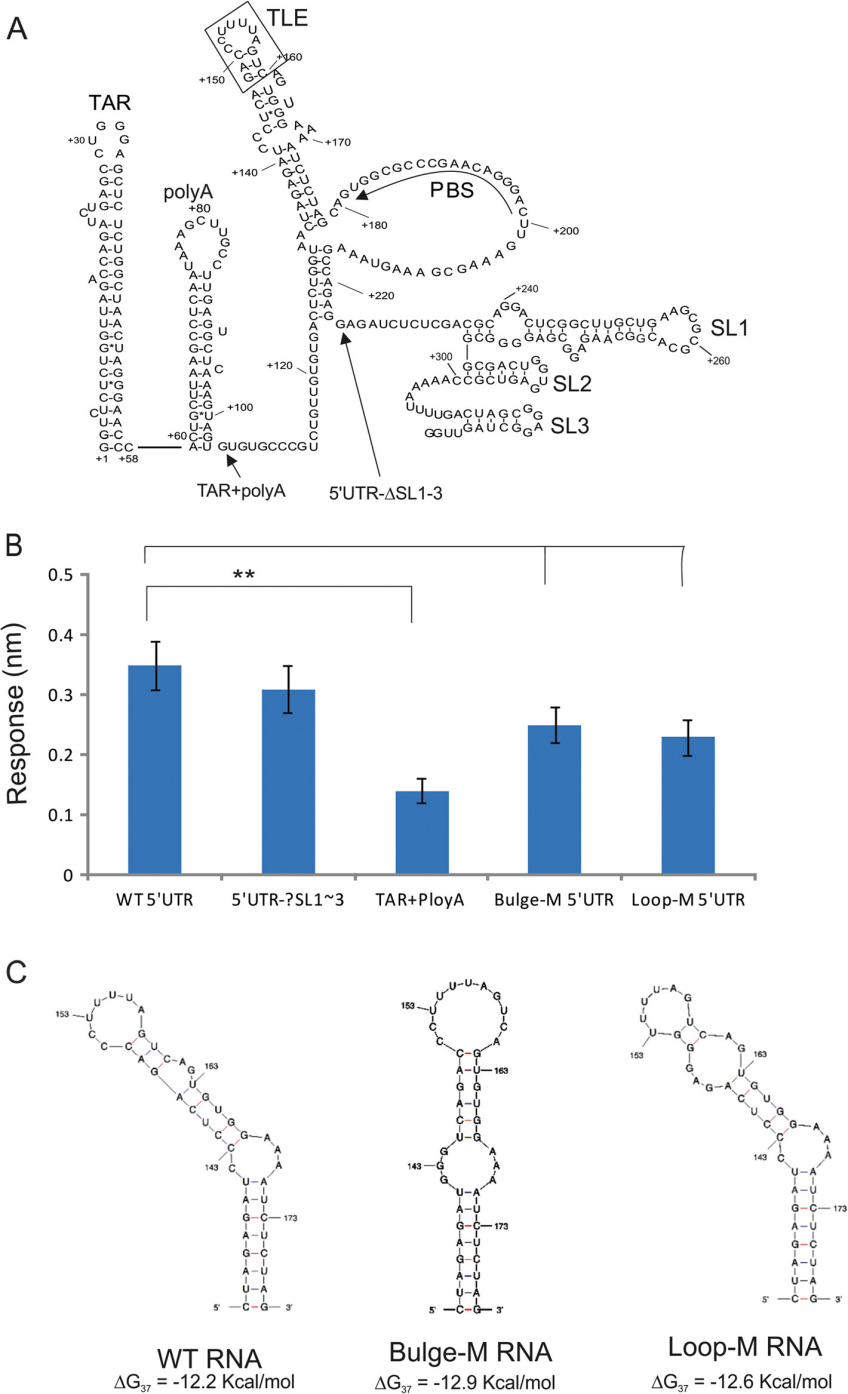
The stem-loop formed by nucleotide 142 to 170 in 5’UTR of HIV-1 genomic RNA is important for interaction with eEF1A. **a** The predicted secondary structure of HIV-1 5’UTR is formed by TAR, polyA, tRNA anticodon-like element (TLE), PBS and stem-loop 1, 2, 3 (SL1, 2, 3). The arrows indicate the sites of truncated RNA. **b** Biotin labelled wild-type, truncated and mutated 5’UTR RNAs were immobilized on biosensors. The maximum responses (nm) during a 600 s incubation of each biosenor with 90 nM of eEF1A protein using OctetRed system are shown. **c** The local secondary structures of wild-type, loop and bulge mutated RNAs and their minimum free energy (ΔG) were predicted using Mfold [21]. All data sets are presented as mean ± SD from at least 3 independent experiments and * indicates *p* < 0.05

5’UTR of HIV-1 genomic RNA contains several well-defined RNA elements with important roles in HIV-1 replication. These elements include TAR, PolyA loop, TLE, PBS, and the stem-loops 1 to 3 (SL1 to SL3) (Fig. 2a) [15, 18]. To better define the eEF1A binding region in the 5’UTR RNA, two truncations of the 5’ UTR RNAs were synthesized. The first truncation was made by removing the stem-loops SL1-SL3 (designated 5’UTR-ΔSL1-3). The second RNA contained only TAR and polyA regions (designated TAR + polyA). The interaction of the truncated 5’UTR RNAs, 5’UTR-ΔSL1-3 and TAR + polyA with eEF1A were examined using BLI assay by immobilizing biotinylated RNAs onto biosensors as described earlier. The 5’UTR-ΔSL1-3 RNA showed a similar binding profile with eEF1A as the intact 5’UTR RNA, and the maximum responses (nm) of the interaction with 90 nM eEF1A is 0.32 nm (no significant difference compared to the intact 5’UTR), whilst the interaction of TAR + polyA with eEF1A was significantly reduced (*p* < 0.05) (Fig. 2b). The results suggest that RNA sequences after polyA and before SL1-3, which includes the TLE and PBS (nt 106 to 224), are important for optimal interaction with eEF1A.

### Alterations of the stem-loop structure that contains TLE in 5’UTR RNA impair eEF1A binding

The region of the viral genome from nt 106 to 224 contains a highly conserved RNA stem-loop structure [17], which contains a TLE [15]. To examine whether this RNA stem-loop is important for the interaction with eEF1A, two mutants of 5’UTR were made in two highly conserved sequence clusters [19, 20]. One mutant was created by changing CCC of nt 142 to 144 in the bulge region to GGG (designated Bulge-M) and another mutant was generated by changing CCC of nt 150 to 152 in the loop region to GGG (designated Loop-M) (Fig. 2a). RNA-folding analysis shows that introductions of these mutations alter the local RNA structures formed by nt 142 to 170 (Fig. 2c) [21]. BLI assay were performed to examine binding of the mutated 5’UTR RNAs with eEF1A protein as described above. The binding of the two mutant 5’UTR RNAs with eEF1A were significant reduced compared to wild-type RNA (*p* < 0.05) (Fig. 2b), indicating that the RNA stem-loop structure formed by nt 142 to 170 are important for binding by eEF1A.

### Association of eEF1A with reverse transcriptase was reduced in cells infected with viruses that contain the bulge and loop mutations

**Fig. 3:**
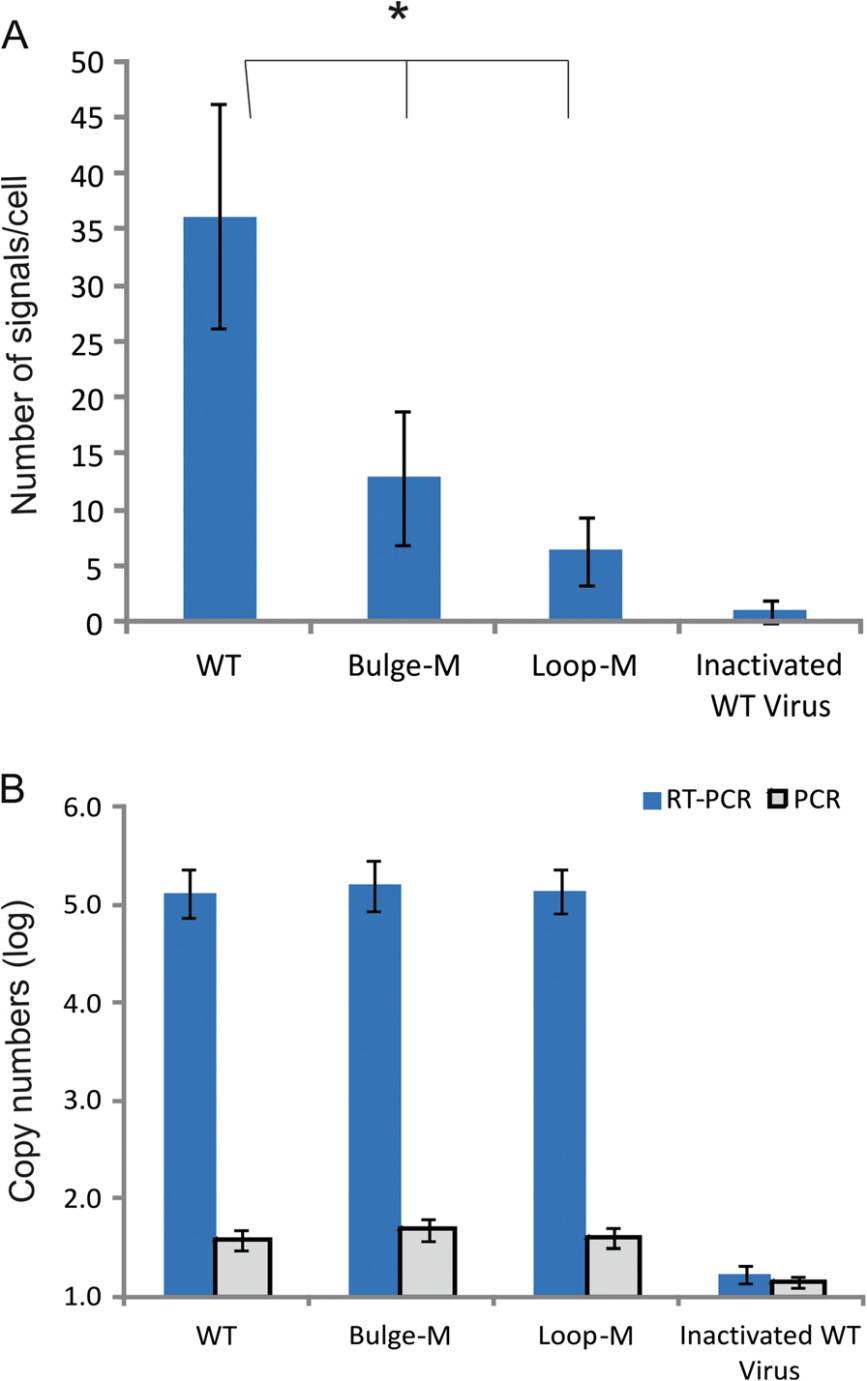
Bulge and loop mutations in 5’UTR of HIV-1 genomic RNA resulted in reduced association of eEF1A and RT in virus infected cells. **a** TZM-bl cells were infected with WT, Loop-M, Bulge-M virus as indicated equal to 100 ng CAp24. Association of eEF1A with HIV-1 RTC in virus infected TZM-bl cells was determined by proximity ligation assay using anti-eEF1A and anti-RT antibodies at 2 h of post-infection. A red foci represents a positive signal that was visualized using a DeltaVision Core imaging system and analysis was performed from more than 200 cells. **b** The levels of genomic RNA at 2 h of post-infection were determined by RT-PCR and possible DNA contamination was examined by PCR without RT

The Bulge-M and Loop-M mutations were introduced into a HIV-1 proviral plasmid DNA and the virus was made by plasmid transfection into HEK293T cells. An equivalent amount of each mutant and wild-type virus, normalised to CAp24 levels, was used to infect TZM-bl cells. Association of eEF1A with HIV-1 RT, a surrogate marker of HIV-1 reverse transcription complex (RTC), was determined by PLA at 2 h post-infection. PLA is a modified fluorescent *in situ* hybridization method to detect protein-protein interactions that produces fluorescent foci if the two PLA antibodies are in proximity. Heat-inactivated virus, which is defective for viral entry, was used to measure non-specific foci made by the PLA antibodies. There was a significant reduction in the number of foci present in cells infected with the mutant compared to wild type virus (*P* < 0.01), while inactivated wild type, which are defective for viral entry, virus produced very few foci (Fig. 3a). The levels of virus entry into cells, as determined by measurement of genomic RNA in the cytoplasm at 2 h of post-infection, were similar indicating that reduced levels of eEF1A-RTC association in mutated virus infection were not due to differences in viral entry (Fig. 3b). As expected, heat-inactivated virus sharply reduced levels of viral RNA in cells. The results suggest that the RNA stem-loop structure altered by the mutations is important for interaction between eEF1A and RT.

### The Bulge-M and Loop-M mutations in 5’UTR of HIV-1 genome affect late steps in HIV-1 reverse transcription

**Fig. 4:**
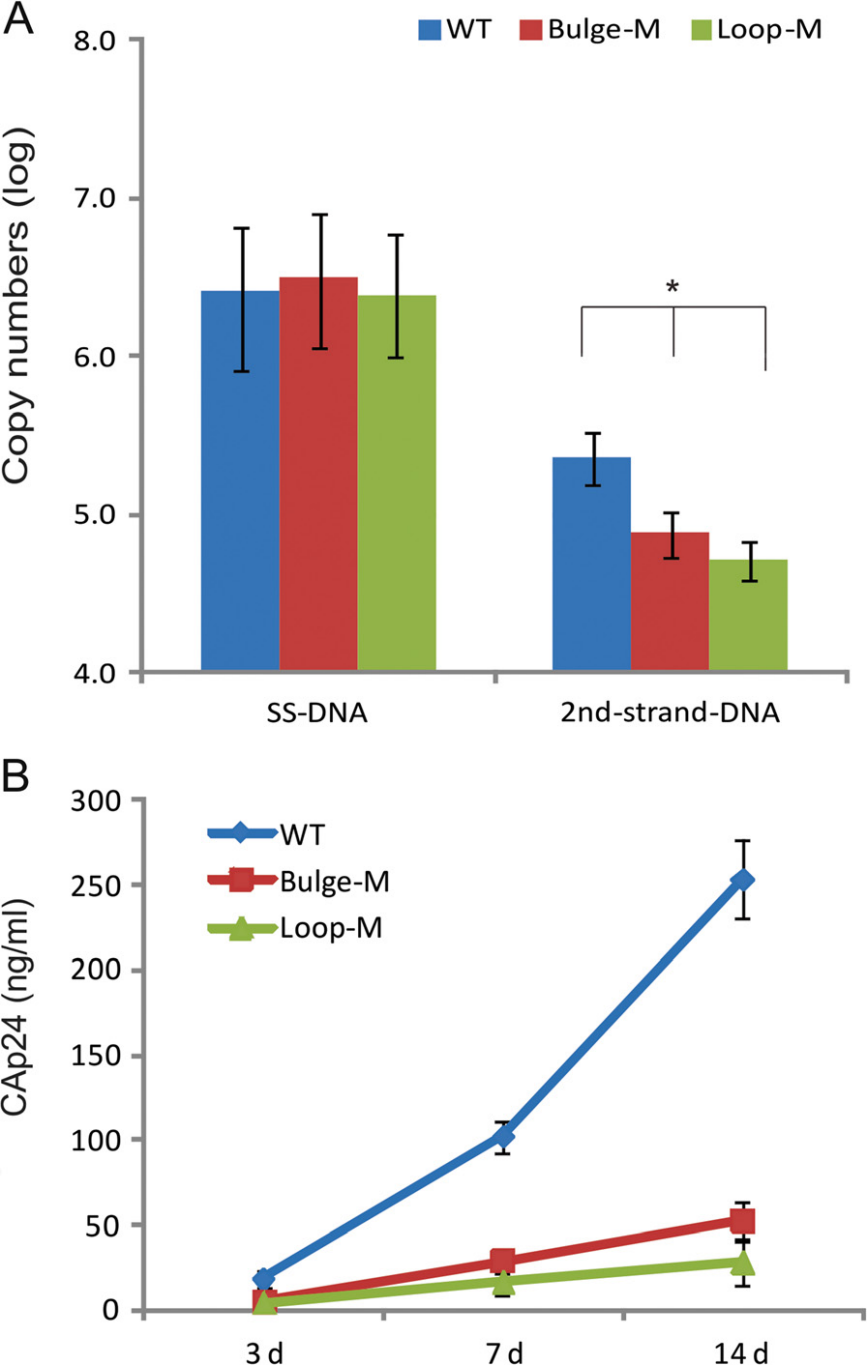
HIV-1 with bulge and loop mutations in 5’UTR of HIV-1 genomic RNA undergo reduced levels of reverse transcription and replication in Jurkat cells. **a** Jurkat cells were infected with equivalent amount of wild type, bulge and loop mutated HIV-1. Viral DNA was extracted from cytoplasm at 4 h post-infection and analysed by quantitative PCR measuring early and late reverse transcription DNA products. **b** Virus replication was monitored for 14 days by measuring CAp24 in culture supernatant at 3, 7 and 14 days post-infection. Data are presented with mean ± SD of 3 independent experiments

The effect of the Bulge-M and Loop-M mutations on reverse transcription and replication was examined by infecting Jurkat cells with equivalent amount of either wild-type or mutant viruses. The total cellular nucleic acids was extracted from cytoplasm at 4 h of post-infection followed by qPCR detection of the early (minus strand strong-stop DNA, ssDNA) and late (2^nd^ strand transfer DNA) reverse transcription products. While the levels of early DNA from the three virus stocks infection were similar (*P* > 0.05), the 5’UTR RNA mutations resulted in a significant reduction in the amount of late RT product detected (*p* < 0.05) (Fig. 4a). The levels of late DNA from the Bulge-M and Loop-M mutated virus infections dropped 3-4 fold compared with wild type virus infection (Fig. 4a). Jurkat cells individually infected with wild type or mutant viruses were monitored for virus replication by measuring CAp24 in culture supernatant for 2 weeks. Both mutant viruses showed a significant reduction in replication kinetics compared to wild type virus (Fig. 4b). The results indicate that the structural changes in the RNA introduced by the two mutations, which affect eEF1A binding to the RNA, lead to reduced efficiency of reverse transcription as observed by reduced levels of late DNA products compared to early DNA products. Hence, the mutations affect the replication of HIV-1 in Jurkat cells.

## Discussion

eEF1A was reported to have multiple roles in many virus replications [1]. Here, we demonstrate, for the first time, that eEF1A can bind to 5’UTR of HIV-1 genomic RNA, which has functional roles in HIV-1 reverse transcription and replication. A stem-loop structure, located at nt 142 to 170 of 5’UTR is important for the binding to eEF1A as well as virus replication.

Recently, a TLE was identified in the HIV-1 5’UTR region of the genomic RNA, formed by nt 148 to160 [15], which overlaps nt 142 to 170 identified in this study. The TLE was shown to interact specifically with human lysyl-tRNA synthetase (hLysRS) and the interaction is important for the efficient annealing of tRNA^lys3^ to viral RNA and virion packaging [15]. Both eEF1A and hLysRS are able to bind tRNA or tRNA like structures, and both are incorporated into HIV-1 virions [13, 22, 23]. As we have demonstrated that eEF1 complex associates with HIV-1 reverse transcription complex [12], it is very likely that eEF1A interacts with genomic RNA during reverse transcription. We showed that clustered mutations in the 5’UTR result in structural changes in the 5’UTR of HIV-1 RNA and reduce binding by eEF1A, which has significant effect on the synthesis of late DNA but not on early DNA products of reverse transcription. These results suggest that while the bulge and loop mutation interfered with association with eEF1A, they did not affect annealing of tRNA^lys3^ to the viral genome because infection of cells with equivalent amounts of wild type, bulge or loop mutant HIV produced early viral DNA at similar levels (Fig. 4). Our results here somewhat mirror our previous study where downregulation of eEF1A by siRNA-treatment in HIV-1 infected cells similarly reduced the late reverse transcription DNA synthesis [12]. Exactly how eEF1A affect late steps reverse transcription through interaction with 5’UTR RNA is unclear. The results here suggest that eEF1A may engage the 5’UTR RNA but subsequently interacts with the RTC by an alternative and presently unknown mechanism.

As an RNA binding protein, eEF1A was reported to bind to various RNA structures in many different RNA viral genomes. The most extensively studied structures are tRNA-like structures (TLS) identified at 3’end of same virus genomic RNAs. For example, early investigations of bacteriophage RNA noted that cloverleaf-shaped tRNA-like structures (TLSs) at the 3’end of RNA phage Qβ binds with EF-Tu, the prokaryotic homolog of eEF1A, to facilitate phage replication [24]. Subsequently, eEF1A1 was shown to bind TLSs in the 3’UTRs of some RNA plant viruses (reviewed in [25]) thereby affecting translation of viral proteins, viral RNA synthesis and facilitation of viral RNA encapsidation [26–28]. eEF1A was also reported to bind to a RNA element referred to as a “replication silencer” element (RSE) in the 3’UTR RNA stem-loop structure of tomato bushy stunt virus (TBSV) genomic RNA [3]. The RSE interaction with eEF1A was shown to stimulate minus-strand RNA synthesis [29]. eEF1A was shown to bind specifically to a conserved stem-loop structure at 3’UTR of West Nile virus (WNV) genomic RNA [30] to facilitate viral minus-strand RNA synthesis [4].

eEF1A was also shown to bind RNA structures outside the 3’UTR of viral genomes. Examples include the right terminal stem-loop in hepatitis delta virus (HDV) genomic RNA and an RNA cloverleaf structure at 5’end of poliovirus genome [1]. The right terminal RNA stem-loop domain of the HDV genome is a 199 nt RNA element that contains a proposed initiation site for HDAg mRNA transcription and negative-strand RNA synthesis [31, 32]. The 5’-terminal 110 nt of the poliovirus RNA genome can fold a secondary structure resembling a cloverleaf and functions to recruit the poliovirus proteinase 3CDpro. The interaction of eEF1A with the cloverleaf structure may be necessary for virus replication [33, 34]. Our study demonstrates that eEF1A can bind to the stem-loop structure in 5’UTR of HIV-1 genomic RNA, and mutations in the RNA that disrupt the structure disrupt association of eEF1A and RT leading to reduced HIV-1 reverse transcription efficiency and virus replication. We propose that the HIV-1 5’UTR interaction with eEF1A plays an important role in facilitating for eEF1A function in reverse transcription. HIV genomic RNA is highly structured throughout [17] and this study focuses on the 5’UTR, whether eEF1A also binds to other parts of the RNA for other roles remain for further investigation.

## Conclusions

We conclude that the eEF1A-5’UTR RNA interaction is important for efficient completion of HIV reverse transcription. As eEF1A also binds to HIV Gag [13], RT [12] and integrase [35] proteins, it is likely that eEF1A plays multiple roles in HIV replication and the implications of these roles warrant further investigation.

## Materials and methods

### Cell lines and virus culture

HEK293T and TZM-bl cell lines were grown in Dulbecco's modified Eagle's medium supplemented with 10 % heat-inactivated newborn bovine serum and 1× penicillin-streptomycin. Jurkat cells were grown in RPMI 1640 supplemented with 10 % newborn bovine serum and penicillin-streptomycin. All cell lines were incubated at 37 °C in 5 % CO_2_. A stock of wild type and mutated HIV-1_NL4.3_ were generated by transfection of the corresponding proviral DNA using Lipofectamine 2000 (Invitrogen, Carlsbad, CA) into HEK293T cells according to the manufacturer's recommendations. Cell culture supernatants were removed at 48 h posttransfection and centrifuged (200 × *g*, 10 min), and the supernatant was filtered (0.45 μm), treated with DNaseI and stored in 1-ml aliquots at −80 °C.

### Plasmid constructs

The pGCH infectious molecular clone is a HIV-1 proviral plasmid which expresses authentic HIV-1 RNA using the CMV immediate early promoter [36, 37]. The shuttle contains a *MluI* (-604, containing the CMV promoter) and *SphI* (+993) fragment from pGCH cloned into pNEB193. Mutations were made using Quikchange site-directed PCR mutagenesis as per manufacturer’s instructions (Stratagene) on the shuttle vectors and then replaced back in the original backbone of pGCH. The bulge structure mutation (Bulge-M) was made by changing CCC sequence at 142-144 nt to GGG and the top loop region mutation (Loop-M) was generated by mutation of CCC at 150-152 nt to GGG.

### Biotin-labelled RNA transcription

DNA fragments contain a sp6 RNA promoter sequence and HIV-1 5’UTR sequence or RT codoning sequence were amplified by PCR using pGCH and its mutants as template. The PCR products were purified and used as template in the *in vitro* transcription reactions in the presence of dUTP-biotin using a sp6 transcription kit (Roche).

### Reversible crosslink co-immunoprecipitation (CR-co-IP)

The experiment was performed as previously described with modification [38] using either HIV-1 infection or biotin-labelled RNA transfection. Briefly, TMZ-bl cells were infected with HIV-1_NL4.3_ virus in the presence/absence of the HIV-1 RT inhibitor nevirapine at the final concentration of 10 μM. To monitor for possible DNA contamination from the virus stock, heat-inactivated virus was used as a negative control. The virus was incubated with cells for 2 h at 4 °C and 2 h at 37 °C and then removed. Alternatively, cells were transfected with biotin-labelled RNA and the cells were collected at 2 h post-transfection. Next, the cells were washed three times with PBS followed by crosslinking using 1 % formaldehyde in PBS for 10 min at room temperature, which was stopped by adding 0.2 M glycine. The cells were washed with PBS once and then lysed in hypotonic buffer containing RNase-free DNaseI, RNase inhibitor and proteinase inhibitors using Dounce homogenization. The lysate was centrifuged at 16000 × *g* at 4 °C for 30 min and the supernatant was incubated with anti-eEF1A antibody coated beads at 4 °C for 4 h followed by three washes with PBS. The final immunoprecipitation (IP) product was resuspended in elution buffer (50 mM Tris-HCl, PH 7.0, 5 mM EDTA, 10 mM DTT and 1 % SDS) and heated for 45 min at 70 °C to reverse the crosslinking. The RNA was extracted using Trizol reagent (Life Technologies, USA) according to the manufacturer’s protocol. HIV-1 RNA was detected by RT-PCR targeting 5’UTR as described previously [39]. DNA contamination was monitored by qPCR (omitting addition of reverse transcriptase). To determine the levels of biotin-labelled RNA, the RNA was loaded on the Hybond membrane (Amersham Bioscience) followed by crosslink using UV light for 3 min. The membrane was then blocked using pierce protein free T20 blocking buffer (Thermo Sciencfic, USA) and incubated with streptavidin-peroxidase (Zeptometrix, USA) for 1 h at room temperature and detected using clarity western ECL substrate (Bio-Rad, USA).

### Biolayer Interferometry (BLI) assay

BLI assay was performed as previous described with some modifications [40] Biotin-labelled RNA was immobilized onto streptavidin coated biosensors (Pall ForteBio, CA, USA) by incubating the biosensors in 1 μM RNA solution for 15 min with 800 rpm shaking in the OctetRed system (Pall ForteBio, CA, USA). The association of RNA with eEF1A protein (Origene Technologies, MD, USA) was measured by incubating RNA biosensors in kinetic buffer (1 mM phosphate, 15 mM NaCl, 0.002 % Tween-20 and 0.1 mg/ml BSA) containing various concentration of eEF1A or eEF1G proteins (analyte) with 1000 rpm shaking in the OctetRed system. The dissociation was determined by moving the ligand biosensor from the analyte solution to kinetic buffer.

### Proximity ligation assays (PLA)

PLA assay was performed as previous described [12]. Briefly, TZM-bl cells were incubated with wild type or mutant virus at 4 °C for 2 h to allow for virus attachment. The cells were then incubated 2 h at 37 °C to initiate virus fusion, viral entry and reverse transcription. The cells were fixed using 4 % paraformaldehye followed by permeabilization by acetone*.* Duolink proximity ligation assays were performed using a mouse monoclonal antibody to detect HIV-1 RT in conjunction with rabbit antibodies to eEF1A (Santa Cruz). DAPI stain was used to visualize the nuclei. Cells were visualized using a DeltaVision Core imaging system. Maximum-intensity projections of deconvolved images were analyzed using Duolink Image Tool software. More than 200 cells from at least 20 fields were analyzed.

### HIV-1 entry assay

TZM-bl cells were incubated with wild type or mutated virus in the presence of nevirapine at 4 °C for 2 h and then at 37 °C for 2 h. The cells were washed for three times and then collected using Trizol® reagent (Life Technologies). The RNA fraction was column purified using a Direct-zol mini-prep kit (Zymo Research) and treated on the column with DNaseI prior to elution. The purified RNAs were used in RT-PCR reactions in the presence or absence of SuperScript® III reverse transcriptase (Life Technologies) using random hexamer oligonucleotides for the first strand DNA synthesis. qPCR was performed as previously described [37].

### CAp24 ELISA

CAp24 antigen in culture supernatant was measured by using a RETROtek HIV-1 Cap24 antigen enzyme-linked immunosorbent assay (ELISA) (Zeptometrix, USA) according to the manufacturer's instructions.

### RNA folding analysis

The RNA secondary structures were predicated using online program (http://mfold.rna.albany.edu) with the conditions, folding temperature of 37°, ionic conditions of 1 M NaCl, no divalent ions; an upper bound number of computed folding was 50; the maximum interior/bulge loop size was 30.
